# Multi-Operational Selective Computer-Assisted Targeting of hepatocellular carcinoma—Evaluation of a novel approach for navigated tumor ablation

**DOI:** 10.1371/journal.pone.0197914

**Published:** 2018-05-23

**Authors:** Pascale Tinguely, Marius Schwalbe, Torsten Fuss, Dominik P. Guensch, Andreas Kohler, Iris Baumgartner, Stefan Weber, Daniel Candinas

**Affiliations:** 1 Department of Visceral Surgery and Medicine, Inselspital, Bern University Hospital, University of Bern, Bern, Switzerland; 2 ARTORG Center for Biomedical Engineering Research, University of Bern, Bern, Switzerland; 3 Department of Clinical and Interventional Angiology, Inselspital, Bern University Hospital, University of Bern, Bern, Switzerland; 4 Department of Anesthesiology and Pain Medicine, Inselspital, Bern University Hospital, University of Bern, Bern, Switzerland; Texas A&M University, UNITED STATES

## Abstract

**Objective:**

To facilitate precise local ablation of hepatocellular carcinoma (HCC) in a setting of combined ablation and transarterial chemoembolization (TACE), we evaluated accuracy and efficiency of a novel technique for navigated positioning of ablation probes using intrahepatic tumor referencing and electromagnetic (EM) guidance, in a porcine model.

**Methods:**

An angiographic wire with integrated EM reference sensor at its tip was inserted via a transarterial femoral access and positioned in the vicinity of artificial liver tumors. The resulting offset distance between the tumor center and the intrahepatic endovascular EM reference was calculated. Subsequently, EM tracked ablation probes were inserted percutaneously and navigated toward the tumor center, relying on continuous EM guidance via the intrahepatic reference. Targeting accuracy was assessed as the Euclidean distance between the tip of the ablation probe and the tumor center (Target Positioning Error, TPE). Procedural efficiency was assessed as time efforts for tumor referencing and tumor targeting.

**Results:**

In 6 animals, 124 targeting measurements were performed with an offset distance < 30 mm (clinically most feasible position), resulting in a mean TPE of 2.9 ± 1.6 mm. No significant correlation between the TPE and different intrahepatic offset distances (range 21 to 61 mm, n = 365) was shown as long as the EM reference was placed within the liver. However, the mean TPE increased when placing the EM reference externally on the animal skin (p < 0.01). TPE was similar when targeting under continuous ventilation or in apnea (p = 0.50). Mean time for tumor referencing and navigated targeting was 6.5 ± 3.8 minutes and 14 ± 8 seconds, respectively.

**Conclusion:**

The proposed technique allows precise and efficient navigated positioning of ablation probes into liver tumors in the animal model. We introduce a simple approach suitable for combined ablation and TACE of HCC in a single treatment session.

## Introduction

Hepatocellular carcinoma (HCC) is the third leading cause of cancer-related death worldwide [1]. However, less than 30% of patients with HCC qualify for standard curative treatments such as complete surgical resection, liver transplantation or local ablation. This is most often due to the extent of tumor burden or the severity of the underlying liver disease.

Image-guided thermal ablation using radiofrequency or microwaves are increasingly performed for patients with early stage HCC and as part of downstaging or bridging strategies in patients awaiting transplantation [2,3]. While long-term outcomes equivalent to surgical resection were shown for ablation of small HCC [4], the difficulty to achieve complete necrosis of larger tumors often leads to unsatisfying results in patients with HCC > 3 cm [5,6]. To augment local tumor control in these patients, transarterial chemoembolization (TACE) has been combined with thermal ablation, taking advantage of the synergistic effects of both treatments [7–9]. They are explained mainly by a reduction of perfusion-mediated tissue cooling and the presence of ischemic edema after TACE, which enhances tumor necrosis during consecutive thermal ablation. Contrarily, satellite lesions which are commonly found around larger lesions and which are difficult to reach by means of ablation, can be controlled by TACE [6]. Several randomized controlled studies and meta-analyses confirmed the benefit of combined treatment strategies over monotherapy, improving overall and recurrence-free survival especially for intermediate- and large-size HCC [8,10–14]. While no consensus has been reached regarding the optimal time interval between both treatments, synergistic effects may be highest when performing both ablation and TACE simultaneously [15,16].

Further important aspects of a successful ablative therapy are accurate tumor visualization and precise placement of ablation probes. In a combined treatment approach in particular, tumor visibility can be altered due to prior chemoembolization [17]. To enhance sensitivity of tumor localization and precision of tumor targeting, advanced computer-assisted navigation technologies have been introduced, and recent studies confirm increased targeting accuracy for image-guided interventions [18,19]. Most navigation techniques rely on image-to-patient registration, the alignment of perioperative image data with the intraoperative patient and organ position. This process is affected by a number of error sources, resulting in potential inaccuracies in the guidance information. Procedures requiring registration of soft tissues are particularly susceptible to inaccuracies over time, due to frequent displacement of the internal target relative to the registered references. These displacements are mostly induced by breathing motion or by tissue deformation during intraoperative manipulation. A possible approach to address this issue is to reduce the distance between the internal target and the registered references to a minimum, potentially leading to synchronous movement of the tracked reference and the target. Adding electromagnetic (EM) navigation technology as the tracking modality for target referencing allows a dynamic scenario, in which the reference position is continuously updated while avoiding inaccuracies associated with an explicit registration process.

To address the challenge of precise thermal ablation in an angiographic setting and thus facilitate a combined therapeutic approach for TACE and ablation in a single treatment session, we propose a technique termed Multi-Operational Selective Computer-Assisted Targeting (MOSCAT) of HCC. This approach combines i) internal tumor tracking using an endovascular intrahepatic reference, taking advantage of the almost exclusive arterial vascularization of HCC and the selective arterial catheterization already performed during TACE, and ii) consecutive percutaneous positioning of tracked ablation probes using dynamic EM-based navigation technology.

In a series of ex vivo experiments, we have previously described stability of EM tracking regarding environmental interferences in the angiography suite, feasibility of the applied image evaluation approach as well as ex vivo targeting accuracy of the proposed technique [20]. In the present work, we aimed to confirm that the MOSCAT for HCC approach allows precise and efficient targeting of intrahepatic tumors in an in vivo setting. To this end, accuracy of ablation probe positioning and procedural efficiency in terms of required time efforts were evaluated in a porcine model.

## Materials and methods

### Animal model and animal care

Experiments were conducted in accordance with the international and local guidelines for care and handling of experimental animals. The study was approved by the Bern veterinary office, the responsible committee on the ethics of animal experiments (license # BE 140/14). Six swine (race: *German Large White)* ranging in weight from 60 to 63 kg were used in this study. Animals were bred at the breeding farm Peter Reber, Oberdettigenstrasse 57, CH-3043 Uettligen, a facility supervised by the Swiss Pig Health Service (SUISAG). SUISAG is the authority responsible for the administration of the herd-book, the carrying-out of performance testing and the estimation of breeding values on behalf of Suisseporcs, the official breeding association of the Swiss pig breeders. Health assessment prior to conduction of the experiments included the evaluation of age-specific size, nutritional status, behavior/activity, body posture, breathing, bristles, color of skin/ears/snout, signs of external injuries, scars, abscesses, swollen joints, secretions from eyes/snout, diarrhea, and interest in food and water. Animals were fed commercial feed (Melior #3411 and whey) three times daily up to 12 hours prior to the experiments, with free access to water up to the time of transport to the University Hospital of Bern on the day of the experiment.

After premedication with intramuscular ketamine 20 mg/kg and xylazine 2 mg/kg, anesthesia was induced with 10–15 mg of intravenous (IV) midazolam and 1 mg IV atropine. Following endotracheal intubation, general anesthesia was maintained with propofol 200–500 mg/h and fentanyl 200–300 μg/h IV as required. Vital parameters were continuously recorded and confined to narrow margins. Mean arterial blood pressure was maintained between 50 and 70 mmHg, heart rate between 60 and 90 beats per minute and end-expiratory partial carbon dioxide pressure between 30 and 40 mmHg. Lactated Ringer's solution was administered for fluid homeostasis, with additional crystalloid fluid and hydroxy-ethyl starch boluses as required.

After preparation of the groin, the external iliac vessels were exposed and an arterial (6 French AVANTI+, Cordis J&J, Milpitia, CA, U.S.) and a venous sheath were inserted. Artificial tumors were created using a mixture of 1% agarose, 10% contrast agent (Iopamidol 300 mg/ml) and distilled water, and shaped to form a diameter of approximately 2 cm using rubber forms. A right subcostal laparotomy was performed to insert the pre-formed artificial tumors, which were fixed between the liver lobes using organic glue. The abdominal incision was closed immediately after insertion of the artificial tumors using a continuous suture.

The animals were euthanized immediately after completion of data acquisition, using 40 mmol/L potassium chloride IV for induction of cardiac arrest, after deepening anesthesia with an additional 200 mg bolus of propofol.

### Experimental material and set-up

Experiments were performed in the angiography suite equipped with a high-resolution angiography system (Artis Zee, Siemens, Erlangen, Germany), allowing direct measurement of distances on fluoroscopic images. Basic angiographic material and contrast media were identical to those used in humans. A 5 French catheter (Cook, Bloomington, IN, USA) was used for catheterization of the hepatic artery after celiac and superior mesenteric arteriography. A 2.7 French microcatheter (Progreat, Terumo Corporation, Tokyo, Japan) was inserted into a segmental or subsegmental hepatic artery and advanced toward an intrahepatic artificial tumor under fluoroscopic guidance.

**Fig 1:**
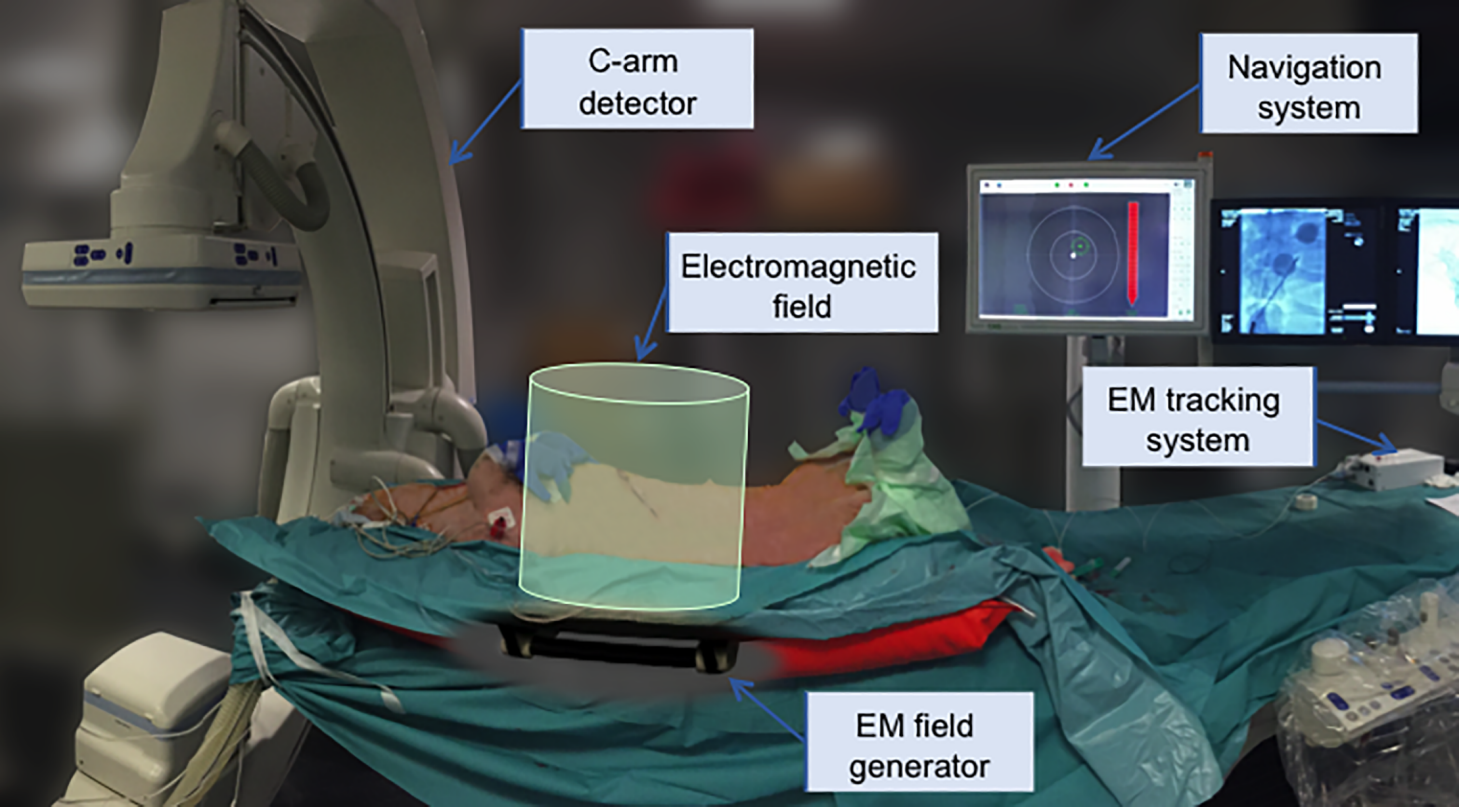
Experimental set-up in the angiography suite. The animal is placed at the level of the EM window field generator attached underneath the carbon angiography table.

A commercially available navigation system (CAS-One Vario, CAScination, Bern, Switzerland) was equipped with EM tracking (Aurora, NDI, Waterloo, Ontario, Canada). The navigation system displays the relative 3D displacement of the targeting device with respect to the chosen target in a cross-hair viewer, with the 3D information separated into orientation and distance. To generate the EM workspace, a window field generator (WFG, NDI Aurora) was attached underneath the angiography table and aligned using a water level. The animal was positioned on the table such that its upper body and liver were covered by the EM field (Fig 1).

**Fig 2:**
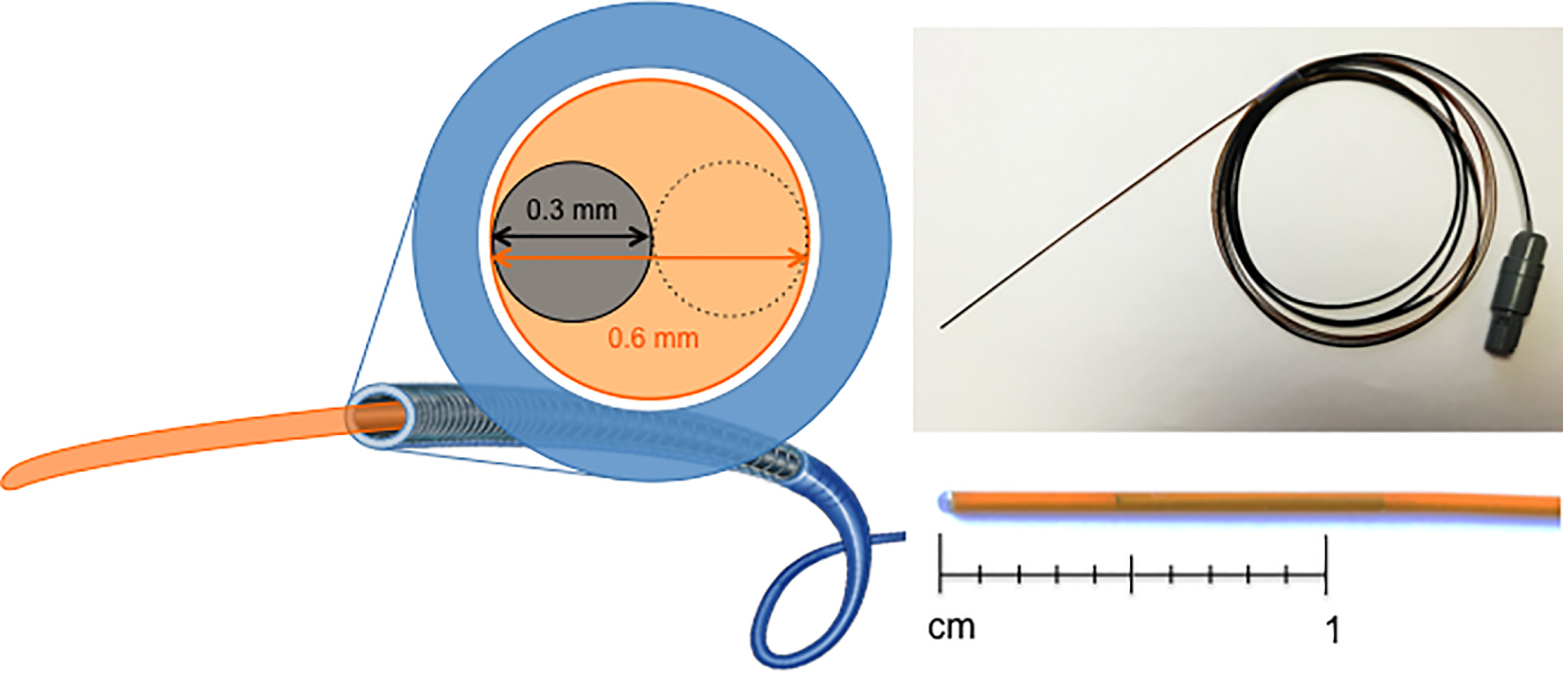
Dimensions and characteristics of prototype angiographic wire with integrated EM reference at the tip. Dimensions: Outer Diameter 0.60 mm, length 140 cm. Characteristics: Polyamide envelope (orange), Stainless steel core (dotted line), EM reference sensor (grey, 0.3 x 8 mm NDI Aurora). Microcatheter (blue, 2.7 French Progreat, Inner diameter 0.65 mm).

To position an EM reference in the vicinity of an intrahepatic tumor in a clinically feasible environment, a prototype of an angiographic wire with integrated EM reference sensor was developed, with corresponding dimensions in order to fit through a 2.7 French microcatheter (inner diameter: 0.65 mm). It consisted of the smallest available EM sensor (5DoF sensor 0.3 mm x 8 mm, NDI Aurora), enforced by a steel core wire and covered by a polyamide tube (outer diameter 0.60 mm, length 140 cm, EPflex Feinwerktechnik GmbH, Dettingen, Germany) (Fig 2).

For percutaneous navigated targeting of intrahepatic tumors, microwave ablation probes (Acculis MTA System, Queensbury, NY, U.S.) were equipped with an externally bonded EM sensor (5DoF sensor 0.5 mm x 8 mm, NDI Aurora).

Both the EM tracked wire and the EM tracked ablation probe were calibrated to their tip by pivoting, a calibration method which estimates the position of the tip as the center of a rotation [21].

### Experimental design

**Fig 3:**
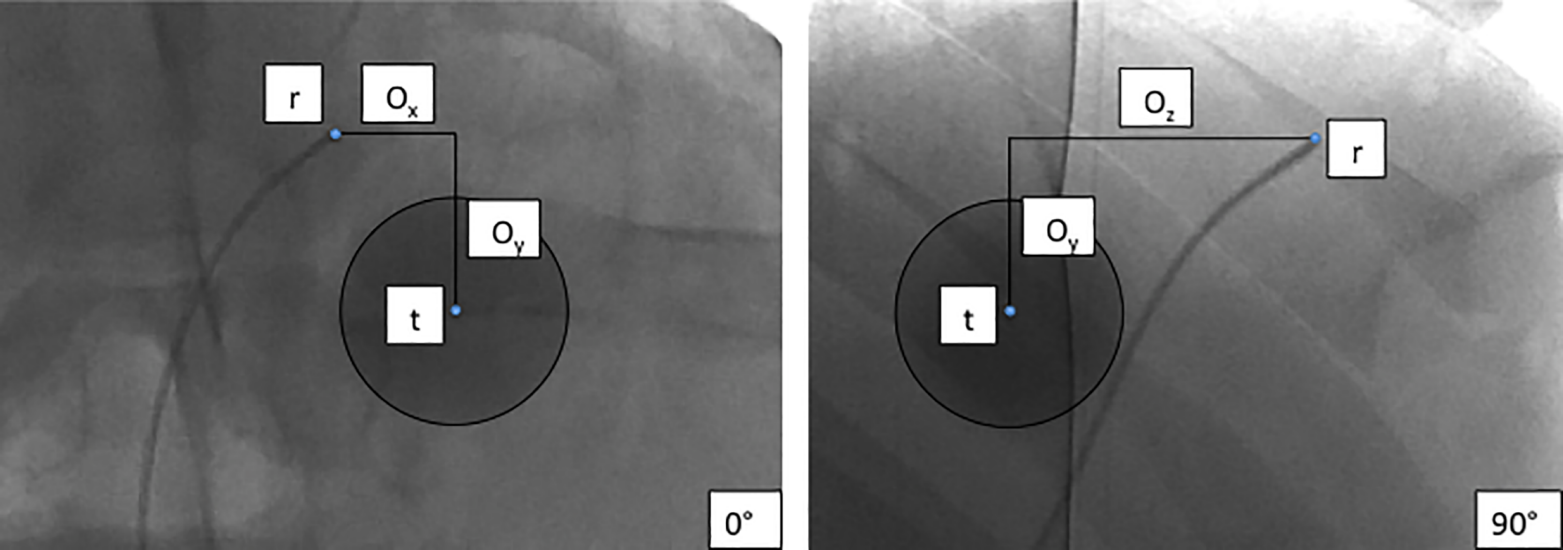
Offset distance measurement. Calculation of the 3D offset vector (o_x,y,z_) from distances between reference sensor (r) and tumor center (target point t), as measured in two perpendicular fluoroscopic images.

The EM tracked wire was inserted into the groin access through the 2.7 French microcatheter and positioned in direct vicinity of an intrahepatic artificial tumor. The resulting offset distance between the tumor and the EM reference mimicked the expected distance when the latter would be placed into tumor supplying arteries of HCC lesions. To assess the position of the tumor center (target point t) relative to the EM reference (r), the distances between the two points were measured in the x, y, and z axes in two perpendicular fluoroscopic images (1024 x 1024 px, pixel spacing 0.25 mm/px) before each targeting series. The target point (t) was calculated in 3D as [*t* = *r* + *o*], where r is the EM reference and o the offset, measured as illustrated in Fig 3. On the navigation system, the intrahepatic target point (t) was then set to these coordinates.

Consecutively, EM tracked ablation probes were inserted percutaneously and navigated toward the intrahepatic target, relying completely on the navigation system for instrument guidance. Separate skin incisions were used for probe insertion to create different angles and lengths of targeting trajectories. During navigated targeting, the C-arm was moved to a maximum distance from the working space to eliminate its influence on the EM field and thus on targeting accuracy [20]. On completion of probe positioning, two perpendicular images of the region of interest were acquired for evaluation of targeting accuracy.

The workflow of the MOSCAT of HCC technique is illustrated in a Video (Video in S1 Video).

**Fig 4:**
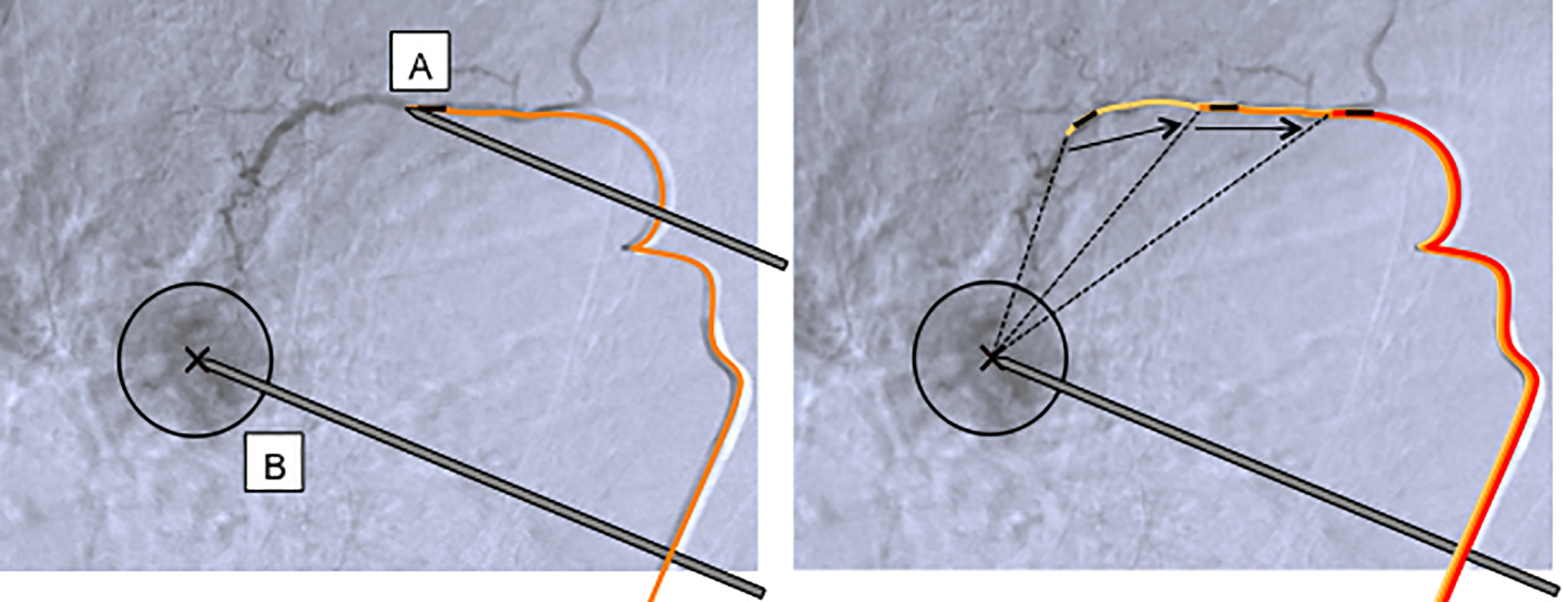
Schematic illustration of targeting measurements. *Left*: baseline (A) and offset (B) targeting measurements, with the EM tracked wire (orange) positioned into a tumor supplying artery. *Right*: Offset measurements at different offset distances between the tumor center and the EM reference (dotted lines), created by pulling back the EM tracked wire inside the hepatic artery (arrows).

Targeting measurements were carried out in two different scenarios (Fig 4, left). To assess targeting accuracy of the proposed navigation technique in a best-case scenario in vivo (direct end-to-end targeting), the ablation probe was aimed directly at the intrahepatic EM reference sensor in a series of baseline measurements. To assess targeting accuracy in a clinically relevant scenario where the EM reference sensor would be placed into tumor supplying arteries of HCC lesions, the ablation probe was aimed at the artificial tumor target, taking into account the calculated offset distance in a series of offset measurements. Different offset distances were created by consecutively pulling back the EM tracked wire by approximately 1 cm (Fig 4, right).

Catheterization of the hepatic artery and positioning of the microcatheter and EM tracked wire under fluoroscopic guidance was performed by an experienced interventional angiologist. Percutaneous navigated positioning of ablation probes was performed by a surgeon experienced with stereotactic ablation of liver tumors.

### Assessments

Targeting accuracy was assessed as the Target Positioning Error (TPE) that resulted after navigated positioning of the ablation probes. TPE was calculated as the Euclidean distance from the tip of the ablation probe to the defined target (EM reference for baseline measurements, center of the artificial tumor for offset measurements). Distances were measured in 3D in two perpendicular fluoroscopic images, using the same approach as described for the offset distance measurement (Fig 3). Matlab (MathWorks, Natick, Massachusetts, USA) was used for manual selection of image points and for calculation of TPE.

Experiments were carried out in six individual animals. As primary endpoint, TPE was assessed when the EM reference was placed in direct vicinity (≤ 30 mm Euclidean distance) relative to the intrahepatic tumor, corresponding to the clinically most relevant position when the EM reference is placed into tumor supplying arteries of HCC lesions (n = 20 in each animal).

As secondary endpoints, factors potentially influencing targeting accuracy were assessed. To this end, TPE was measured i) with the EM reference at five different distances relative to the artificial tumor, to evaluate the influence of the offset distance magnitude on targeting accuracy (n = 50 in each animal), ii) with the EM reference placed on the animal skin, to evaluate the effect when positioning the EM reference at an external location (n = 10 in each animal), and iii) when targeting in complete apnea versus under continuous positive pressure ventilation, to evaluate the influence of the applied breathing modality on targeting accuracy (explicitly assessed in one experimental animal).

Procedural efficiency of the technique was assessed as the time required for i) tumor referencing (from inguinal introduction of the EM tracked wire until reaching the desired position in the vicinity of an artificial tumor), ii) offset measurement and calculation (measurement of the 3D offset distance in fluoroscopic images before each offset targeting series), and iii) tumor targeting (from skin incision and introduction of the tracked ablation probe until the final position in the center of the tumor was reached).

### Statistical evaluation

For assessment of the primary endpoint, a sample size of 100 targeting measurements was calculated based on accuracy measurements conducted in preliminary in vivo experiments (TPE = 2.6 ± 0.7 mm, aiming for a TPE of maximum 3 mm, power of 90%). Linear regression analysis was used to estimate correlation between targeting accuracy and offset distance. The unpaired student’s *t* test was used for between-group analyses of intra- versus extrahepatic position of the EM reference and for different breathing modalities with respect to targeting accuracy. *P* values < 0.05 were considered statistically significant. GraphPad Prism V 7.0 (GraphPad Software Inc, La Jolla, CA, USA) was used for statistical analyses and generation of graphs.

## Results

### Feasibility of the experimental model

All experiments were conducted as planned, with preliminary termination of targeting measurements in one animal due to increased blood loss from the hepatic puncture sites. Mean diameter of pre-formed artificial tumors was 23.1 ± 8.7 mm, as assessed in fluoroscopic images.

**Fig 5:**
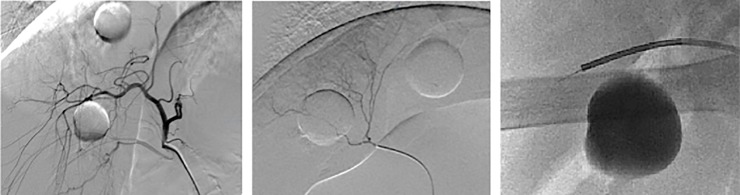
Positioning of EM tracked wire using fluoroscopic guidance. *Left*: Fluoroscopic survey of the porcine right hepatic artery. *Middle*: Selective catheterization of a subsegmental artery close to an intrahepatic artificial tumor. *Right*: EM tracked wire positioned through the microcatheter in direct vicinity to the artificial tumor.

After selective catheterization of a segmental or subsegmental hepatic artery and positioning of the microcatheter under fluoroscopy guidance, the EM tracked wire was successfully positioned in the vicinity of an intrahepatic artificial tumor in all six animals (Fig 5). No loss of signal from the EM sensors integrated into the tracked angiographic wires occurred, and the applied prototypes remained intact throughout the experiments.

### Targeting accuracy

A total of 505 targeting measurements were performed in six animals, consisting of 90 baseline measurements and 415 offset measurements. Of the offset measurements, 124 measurements yielded an offset distance of ≤ 30 mm between the center of the artificial tumor and the EM reference (i.e. primary endpoint).

The relevant results for targeting accuracy and procedural outcomes are summarized in Table 1.

**Table 1:** Targeting accuracy and procedural efficiency of the MOSCAT of HCC technique.

**Targeting accuracy**	TPE, mm	n	Offset distance, mm
Baseline measurements	2.6 ± 1.6	90	n/a
Offset measurements		415	
EM reference within liver	3.2 ± 1.6	365	36.1 ± 11.2
Offset distance ≤ 30 mm (PE)	2.9 ±1.6	124	24.5 ± 2.1
Offset distance > 30 mm	3.3 ± 1.6	241	41.4 ± 9.8
EM reference externally on skin	5.6 ± 2.3	50	102.1 ± 22.5
**Procedural efficieny**	Duration		
Time for tumor referencing (minutes)	6.5 ± 3.8	6	
Time for offset calculation (minutes)	5.2 ± 1.3	35	
Time for navigated targeting (seconds)	13.4 ± 8.3	505	

Distances correspond to Euclidean values in millimeters. Mean ± standard deviations are shown. EM = Electromagnetic, PE = Primary Endpoint, TPE = Target Positioning Error, n/a = not applicable

Mean TPE for baseline measurements was 2.6 ± 1.6 mm (n = 90). Mean TPE for offset measurements with an offset distance ≤ 30 mm (primary endpoint) was 2.9 ± 1.6 mm (n = 124), with a mean offset distance between the center of the tumor and the EM reference of 24.5 ± 2.1 mm.

**Fig 6:**
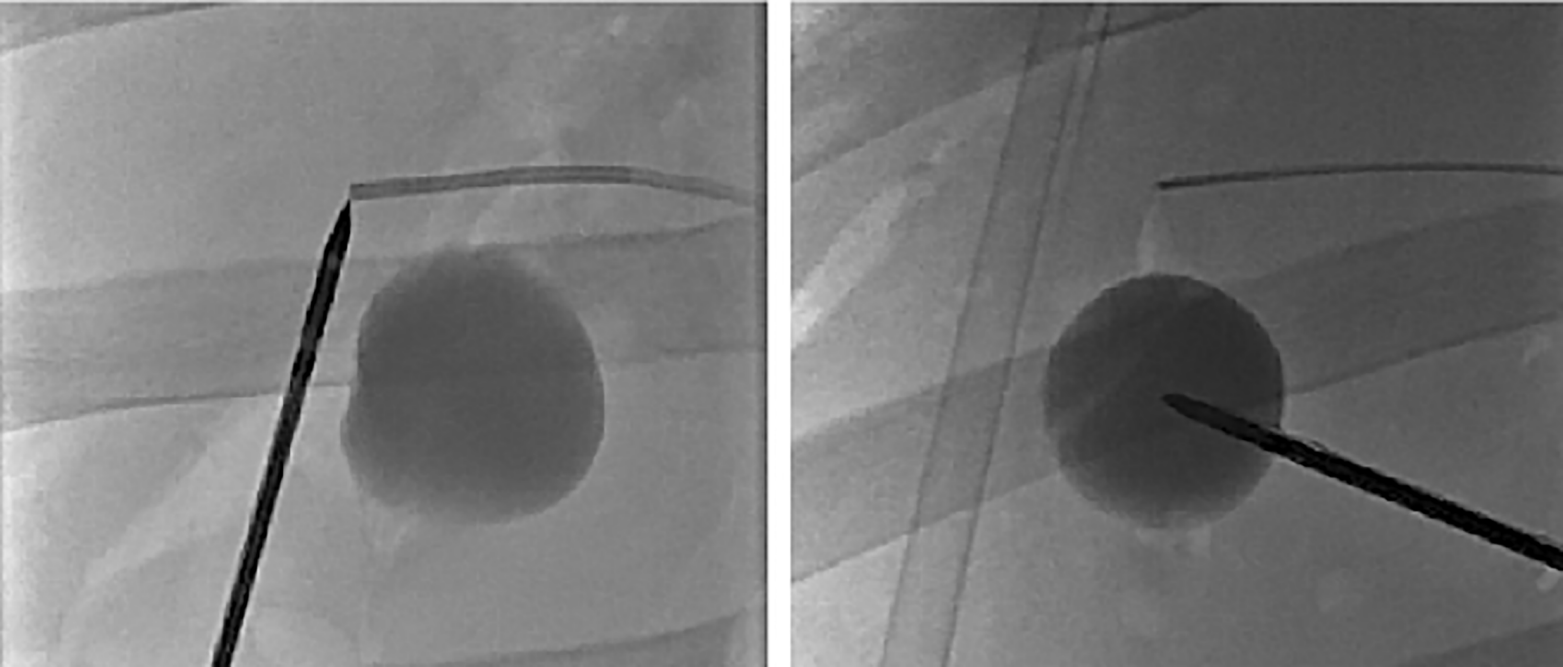
Position of tracked ablation probe after completion of navigated targeting. *Left*: Baseline targeting measurement. *Right*: Offset targeting measurement.

Exemplary results of baseline and offset targeting measurements are shown in Fig 6.

**Fig 7:**
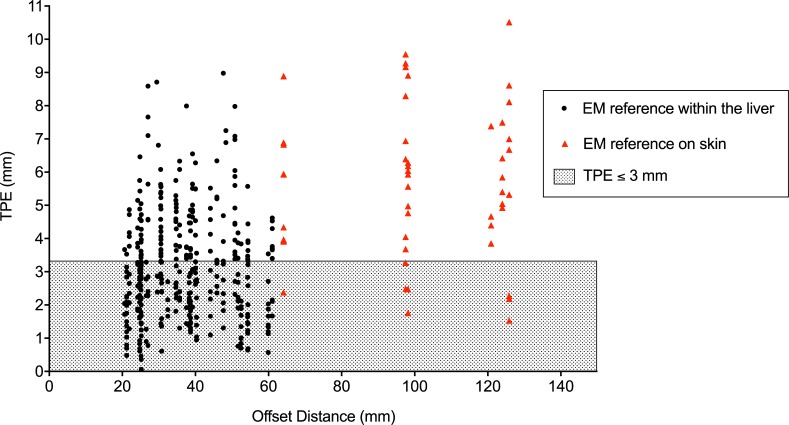
Targeting accuracy according to offset distance. TPE = Target Positioning Error.

When analyzing all offset measurements performed with the EM reference positioned within the liver at different offset distances relative to the artificial tumor (range: 20.6 mm to 61.0 mm), mean TPE was 3.2 ± 1.6 mm (n = 365). No significant correlation was observed between targeting accuracy and the magnitude of the offset distance, as long as the EM reference remained within the liver (r = 0.03, p = 0.62). However, mean TPE increased to 5.6 ± 2.3 mm when the EM reference was placed externally on the animal skin, with a mean offset distance of 102.1 ± 22.5 mm (n = 50, p < 0.01). Accordingly, the rate of targeting measurements that resulted in a TPE of ≤ 3 mm was 52% when the EM reference was positioned within the liver versus 16% when the EM reference was located externally on the skin (Fig 7).

**Fig 8:**
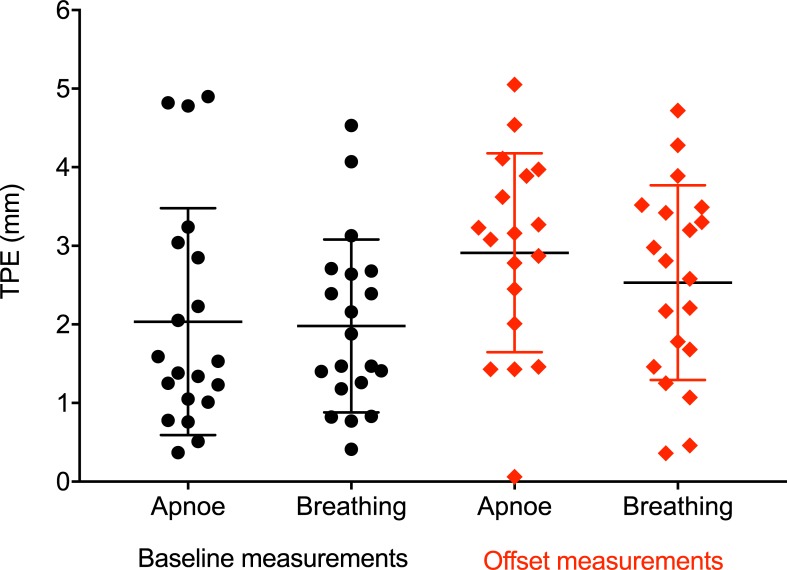
Targeting accuracy according to breathing modality. Bars indicate means and standard deviations. TPE_PE_ = Target Positioning Error, Primary Endpoint.

In the first five animals, navigated targeting was performed in apnea or with intermittent ventilations between the targeting procedure and obtaining fluoroscopic images for image evaluation. In the sixth experimental animal, the influence of different breathing modalities on targeting accuracy was explicitly assessed. To this end, both baseline and offset targeting measurements were performed either in apnea or under continuous ventilation, with 20 measurements in each group. Mean TPE when targeting in apnea versus under continuous ventilation was 2.0 ± 1.4 mm and 2.0 ± 1.1 mm for baseline measurements (p = 0.91), and 2.9 ± 1.3 mm and 2.5 ± 1.2 mm for offset measurements (p = 0.50), respectively (Fig 8).

A displacement of the artificial tumor relative to the surrounding liver tissue and thus to the EM sensor was noticed during the insertion of the ablation probe. This "tumor shift" was 2.2 ± 1.5 mm on average for targeting measurements with an offset distance ≤ 30 mm, and 2.7 ± 2.4 mm for measurements with an offset distance > 30 mm with the EM reference inside the liver.

### Procedural efficiency

Mean time for positioning of the EM tracked wire with integrated EM reference was 6.5 ± 3.8 minutes (n = 6). Mean time for assessment and calculation of the 3D offset from two 2D fluoroscopic images was 5.2 ± 1.3 minutes. Mean time for navigated positioning of ablation probes (from skin incision to final position of EM tracked ablation probe) was 13.4 ± 8.3 seconds (n = 505).

## Discussion

This study confirms accuracy and efficiency of a novel approach for minimally invasive navigated targeting of intrahepatic tumors, in a porcine model. To our knowledge this is the first work describing the combination of an angiographic approach for intrahepatic tumor referencing with percutaneous navigated targeting and positioning of ablation probes.

The potential benefits of using stereotactic navigation systems for targeting and ablation of liver tumors as compared to traditional ultrasound (US) or computed tomography (CT)-guidance include a more accurate and efficient targeting, with a reduction of radiation doses in some settings [18,19,22]. To date, various navigation systems are commercially available for percutaneous image-guided targeting of liver lesions. These systems mostly rely on image-to-patient registration using external or internal reference markers, with either optical or EM tracking technology for instrument guidance [23]. EM tracking technology has emerged for use in the medical field as it introduces several substantial advantages for instrument navigation [24]. Firstly, rigid instruments (e.g. ablation probes) can be tracked at their tip, eliminating potential errors due to instrument bending and the requirement of a direct line of sight [25]. Secondly, flexible instruments such as catheters can be tracked and used for continuous referencing of internal targets such as intrahepatic tumors, when these are made accessible. Combining both of these advantages, the MOSCAT of HCC technique allows a direct instrument-to-target navigation, without the need for potentially erroneous and time-consuming image-to-patient registration. It rather operates within the available dynamic angiographic imaging space, utilizing the tumor's own abundant vascularization for its internal referencing and ultimately its destruction.

A large body of research quantifies the technical performance of electromagnetic tracking systems in the medical field [24]. In the published literature, spatial accuracy of EM-tracking systems is well below one millimeter and is subject to the influence of ferromagnetic materials in the working volume [26]. In the context of image-based hepatic ablation and biopsy, various EM navigation approaches based on registration of different intraoperative imaging modalities with pre-procedural image data have been proposed [27–32]. The reported effective end-to-end targeting errors range between 3–8 mm for in vitro and in vivo measurements [27,29,32,33]. We have previously reported target positioning errors below 3 mm when applying the herein proposed navigation approach in an ex vivo setting [20], and now showed similar targeting accuracies of 2.6 ± 1.6 mm for baseline measurements in vivo. This represents the best possible end-to-end accuracy when applying the proposed technique in an angiographic setting, and thus describes the inherent errors when using EM tracking in this environment. Taking the technique one step closer to a possible clinical application, we now added an offset distance between the EM reference and the tumor target, mimicking the placement of the EM reference into peritumoral vascularization. When including this offset in the current targeting measurements in an in vivo setting, we showed targeting errors of 2.9 ± 1.6 mm. Considering an intended ablation margin of generally 5–10 mm[3] and compared to the targeting accuracies reported in other works, these targeting errors seem favorable and acceptable in a clinical context.

The calculated average displacement of the tumor target relative to the internal reference sensor ("tumor shift") of 2.2 ± 1.5 mm might be due to local tissue compression during the insertion of the rigid ablation probe. In our experimental model this might also be influenced by a tumor displacement due to a less solid fixation of artificial tumors in the surrounding liver parenchyma than in real liver tumors. When excluding targeting measurements with a tumor shift > 3.7 mm (mean tumor shift + 1 SD), mean TPE was reduced to 2.7 ± 1.4 mm (n = 107).

The MOSCAT of HCC technique utilizes a continuous internal tracking of the tumor close to its true location, introducing several hypothetical advantages which we verified and confirmed in this work. Firstly, a synchronous movement of the tumor target relative to the intrahepatic tracking reference might be assumed during motion of the whole organ (e.g. due to breathing). In our series, no significant loss of targeting accuracy was shown when performing navigated targeting under continuous ventilation as compared to targeting in apnea (p = 0.50). The importance of this finding is emphasized when considering the substantial efforts performed to overcome the issue of organ motion in soft tissue navigation are considered. In most reports this involves measuring and adapting for organ motion using complex algorithms [23,27,34]. Secondly, the assumed synchronous movement of the tumor target and the internal tracking reference may explain why there was no difference in targeting errors for different offset distances, as long as the reference sensor remained within the liver (Fig 7). This suggests that the margin of acceptable offset distances between the reference sensor and the tumor target may be relatively wide (up to 6 cm in our series), as long as the reference sensor can be positioned within the liver. This is of importance as the tumor supplying arteries of HCC often have a curled and kinked pathological morphology, making them difficult to access with the routinely applied angiographic catheters and even more so with the EM tracked angiographic wire. Furthermore, this tolerated offset distance might allow targeting of several intrahepatic tumors for ablative treatment with a single placement of an EM reference sensor.

Frequent barriers to introducing novel navigation techniques into clinical practice are the associated technical complexity, additional procedural efforts and cost investments [23,35]. We deliberately aimed to develop a precise, yet simple approach for minimally invasive ablation of HCC in order to minimize the complexity of the technique. The relevant procedural steps of the proposed approach include the positioning of the EM reference sensor into an intrahepatic artery through an installed microcatheter and the measurement of a 3D offset from two 2D fluoroscopic images. In our animal model, these two steps together required a total time of approximately 11 minutes, with consecutive rapid and precise positioning of ablation probes. Furthermore, radiation doses applied for navigated targeting of liver tumors are reduced to an absolute minimum with the proposed technique, requiring 2 single perpendicular fluoroscopic images for measurement and calculation of the offset distance between the tumor and the EM reference.

Most studies reporting a combined treatment approach of TACE and ablation use US or CT imaging with or without fluoroscopic guidance for placement of ablation probes [14,36,37]. However, additional aspects regarding tumor visualization for precise placement of ablation probes must be considered in this setting. Variable uptake of iodized oil and chemotherapeutic agents in and around the tumor can alter tumor visibility in the US image after prior chemoembolization [17]. In addition, tumors > 3 cm often require multiple overlapping ablations, which are technically difficult to achieve with US guidance due to echogenic artifacts. However, retained iodized oil can facilitate tumor visualization in fluoroscopy [15,38], and a recent work showed promising results when using dual guidance of biplane fluoroscopy and US for a combined treatment of TACE and ablation [15].

The MOSCAT of HCC technique is potentially an efficient method to combine TACE and ablation in a single treatment session. As we applied the same angiographic material as clinically used for TACE, a relatively simple combination of both treatment approaches is imaginable. Hypothetically, the sequence would include the positioning of the microcatheter into a suitable position for TACE under fluoroscopic guidance, followed by introduction of the EM tracked wire and percutaneous navigated positioning of the ablation probe. Before applying thermal energy for ablative therapy, the EM tracked wire would be removed such that TACE could be performed according to the standard techniques, with the microcatheter already in place. Then, thermal ablation over the precisely positioned ablation probe could be initiated. Both local destructive treatments would thus be integrated and combined to a single and efficient minimally invasive treatment approach for HCC.

With regard to an evaluation of the proposed technique in a clinical setting, several issues must be considered. Firstly, the MOSCAT of HCC technique focuses on a tumor-directed navigation without registration of surface structures. Hence, additional US guidance is required to control the trajectory of the ablation probe between skin insertion and the intrahepatic tumor [15,38]. In our experimental model, multiple hepatic punctures were performed in each animal without track ablation. Due to this enhanced risk of bleeding and the limited postprocedural observation time, no conclusions regarding safety of the technique can be drawn at this stage. Secondly, the navigation instruments applied in this study are not readily available. While various approaches toward the integration of EM sensors into catheters and wires for endovascular procedures have been described [35,39–42], no EM tracked angiographic device fulfilling the requirements and dimensions for placement into intrahepatic arteries are available to date. We are currently developing and optimizing the prototype of the EM tracked angiographic wire for potential clinical use. A primary concern that must be addressed is the inherent stiffness of the wire tip resulting from the integrated straight 8 mm EM sensor, rendering its positioning into curved and kinked pathologic tumor feeding vessels difficult. Additionally, no EM tracked device for thermal ablation is currently available, although the advantage of using ablation probes tracked at their tip have been reported ex vivo and in vivo [29,43,44]. However, other EM tracked devices such as EM tracked trocars would be readily available for clinical use and have shown similar targeting accuracies in our ex vivo experiments [20]. Ultimately, this study did not include any aspects regarding local ablative treatment or outcomes beyond tumor targeting, and focused solely on the accuracy and efficiency of ablation probe positioning. However, many other factors contribute to a successful local ablation of liver tumors aside from accurate tumor targeting. These include factors affecting the local distribution of thermal energy such as properties of the tumor and underlying liver tissue [45], vessel proximity, and the applied ablation modality, energy and duration. In a future clinical trial, analysis of treatment success after thermal ablation when using the proposed technique for tumor targeting, as well as safety of the procedure and the comparison of the technique to traditional non-navigated image-guidance, must be included.

In conclusion, the MOSCAT of HCC technique allows precise and efficient positioning of ablation probes into intrahepatic tumors in a porcine model. Operating in the available angiographic imaging space, the technique allows dynamic instrument guidance for tumor targeting due to continuous intrahepatic endovascular tumor referencing. We introduce a simple approach for combined TACE and ablation in one treatment session, for selected patients with HCC. Clinical applicability and the impact of the technique on treatment outcome for HCC will be included in a future clinical trial.

## Supporting information

S1 Video Workflow of the MOSCAT of HCC technique. (MOV)
